# Multilevel and multifaceted brain response features in spiking, ERP and ERD: experimental observation and simultaneous generation in a neuronal network model with excitation–inhibition balance

**DOI:** 10.1007/s11571-022-09889-w

**Published:** 2022-11-23

**Authors:** Guang Ouyang, Shengjun Wang, Mianxin Liu, Mingsha Zhang, Changsong Zhou

**Affiliations:** 1 Faculty of Education, The University of Hong Kong, Pok Fu Lam, Hong Kong China; 2 Department of Physics, Centre for Nonlinear Studies and Beijing-Hong Kong-Singapore Joint Centre for Nonlinear and Complex Systems (Hong Kong), Institute of Computational and Theoretical Studies, Hong Kong Baptist University, Kowloon Tong, Hong Kong China; 3 Department of Physics, Shaanxi Normal University, Xi’an, 710119 China; 4 State Key Laboratory of Cognitive Neuroscience and Learning, Beijing Normal University, Beijing, 100875 China

**Keywords:** Excitation–inhibition (E–I) balance, Self-organized criticality, Multilevel neural activity, Nonlinear neural dynamics, ERP and ERD

## Abstract

Brain as a dynamic system responds to stimulations with specific patterns affected by its inherent ongoing dynamics. The patterns are manifested across different levels of organization—from spiking activity of neurons to collective oscillations in local field potential (LFP) and electroencephalogram (EEG). The multilevel and multifaceted response activities show patterns seemingly distinct and non-comparable from each other, but they should be coherently related because they are generated from the same underlying neural dynamic system. A coherent understanding of the interrelationships between different levels/aspects of activity features is important for understanding the complex brain functions. Here, based on analysis of data from human EEG, monkey LFP and neuronal spiking, we demonstrated that the brain response activities from different levels of neural system are highly coherent: the external stimulus simultaneously generated event-related potentials, event-related desynchronization, and variation in neuronal spiking activities that precisely match with each other in the temporal unfolding. Based on a biologically plausible but generic network of conductance-based integrate-and-fire excitatory and inhibitory neurons with dense connections, we showed that the multiple key features can be simultaneously produced at critical dynamical regimes supported by excitation–inhibition (E–I) balance. The elucidation of the inherent coherency of various neural response activities and demonstration of a simple dynamical neural circuit system having the ability to simultaneously produce multiple features suggest the plausibility of understanding high-level brain function and cognition from elementary and generic neuronal dynamics.

**Supplementary Information:**

The online version contains supplementary material available at 10.1007/s11571-022-09889-w.

## Introduction

To study the behavioral pattern of an object and the laws governing it, a common approach in physics is to stimulate the object and observe its response. Similarly, the most fundamental way to study the brain is to give it an input stimulus and observe its response. But the brain is a complex system with multiple levels of organization generating response activity: (1) it is multifaceted and hierarchical, and (2) it displays dominantly nonlinear features (Breakspear 2017; Jia and Gu 2019). Therefore, neural activity at different levels and aspects should be studied integratively to understand how they come about from a unified system; for instance, how the collective neuronal spike activity translates to local field potentials (LFP) and further to scalp potentials, and how different aspects of features (e.g., oscillation, transient dynamic pattern, scale-free dynamics) are linked to each other. Furthermore, we need to recognize the importance of ongoing neural dynamics and how they interact with stimulus inputs and generate considerable amount of neural variability (Dinstein et al. 2015; Faisal et al. 2008; Li et al. 2021a; Kang et al. 2021; Jin et al. 2022), which is functionally relevant (Gonen-Yaacovi et al. 2016; Saville et al. 2015; Peng et al. 2014).

The majority of the previous theoretical and experimental research focused on activity features at a specific level or aspect, such as the phenomenon of neural avalanches, oscillation, and network connectivity patterns (Wang 2010; Chialvo 2010; Rubin et al. 2015; Li et al. 2018). As above-mentioned, these single-level approaches are inherently limited in studying the complexity of the brain. One approach to deepening the understanding of the complexity of the brain and the functions of the multilevel, multifaceted activity and nonlinear effects is to build up generative model simulating the bottom-level activity (i.e., neuronal activity) while being able to reproduce a coherent set of multilevel and multifaceted features in response to external stimulations. Undoubtedly, these models must be built on crucial nonlinear mechanisms in order to reproduce key features of brain dynamics. The self-organized complex dynamic network system in the brain generates complex signals at all levels, including irregular spiking of neurons with statistics close to Poisson random process (Softky and Koch 1993), the scale-free spectrum of cortical electrophysiological signals (Linkenkaer-Hansen et al. 2001; Miller et al. 2009; He 2014), the distinct oscillations amidst the background noise and scale-free activity (Buzsaki 2006; Cole and Voytek 2017; Donoghue et al. 2020; Ouyang et al. 2020), the scale-free neuronal avalanches (Beggs and Plenz 2003; Gireesh and Plenz 2008; Petermann et al. 2009; Bellay et al. 2015), all of which are shown in the resting state brain. These complex and highly nonlinear neural activity patterns generated by the underlying nonlinear dynamical neural network system have been considered to be crucially shaped by generic dynamic mechanisms. One of them is excitation–inhibition (E–I) balance. It has been theoretically demonstrated that E–I balance is the origin of temporally irregular spiking patterns and allows the network to react quickly and linearly to external stimuli (Vreeswijk and Sompolinsky 1996). The balance between excitation and inhibition produces highly variable interspike intervals consistent with experimental data (Shadlen and Newsome 1998), enables effective cortical activity amplifications (Douglas et al. 1995), produces self-sustained oscillatory activity (Wang et al. 2011; Compte et al. 2003) in a way that the rhythm is determined by E–I balance (Brunel and Wang 2003). Besides the theoretical works, the importance of E–I balance has also been shown in many experimental data. The proportional balance between excitation and inhibition was shown to be functionally important in spontaneous activity from both in vitro and in vivo experiments (Shu et al. 2003; Haider et al. 2006), and response activity (Wehr and Zador 2003; Xue et al. 2014). These theoretical and experimental research shows that E–I balance may serve as a generic organization principle of local circuits that determines the self-consistent characteristics of multilevel and multifaceted neural activities.

Another factor that has been commonly proposed to be a key factor supporting the specific and coherent multilevel and multifaceted dynamic patterns is self-organized criticality (SOC). SOC refers to the phenomenon of a dynamical system having an inherent capacity to self-organize itself to a specific critical state regardless of initial conditions (Bak et al. 1987). The specific critical state typically displays spatial or temporal scale-invariance characteristic (Sornette 2006). SOC has been proposed to be a fundamental property of neural systems (Hesse and Gross 2014). Functionally, SOC has also been frequently reported to play an important role in neural information processing (Shew et al. 2009, 2011; Shriki and Yellin 2016).

Given the functional importance of the E–I balance neural architecture and criticality as described above, it may be hypothesized that these two factors play the central roles in the genesis of specific and coherent multilevel and multifaceted dynamic patterns in the neural dynamic system. Noticeably, the previous modelling works by Yang et al. (2017) and Liang and Zhou (2022), Liang et al. (2020) have demonstrated that a biologically plausible E–I balanced networks working at the critical regimes can reconcile irregular spiking, critical avalanche and collective oscillations in the E–I balanced network model. In the presence of external stimulations (Liang and Zhou 2022), the model in the critical regime can further produced several specific neural activity features across multiple levels found in experimental data. These features include (1) high variability in the spontaneous dynamic activity, (2) reduced variability after stimulus input, (3) frequency shifting of ongoing neural oscillation, and (4) preservation of criticality during stimulus processing.

In light of these recent progresses on explaining the multilevel complex features based on E–I balance and criticality, here we further ask the question of whether such generic principle can explain other salient multilevel features—specifically, the co-occurrence of event-related potentials (ERP), event-related desynchronization (ERD), spiking activity dynamics and the coherency amongst them (e.g. timing of the dynamic unfolding). Conventionally, both ERP and ERD have been extensively studied but largely separate from each other (Luck and Kappenman 2011; Luck 2014; Keil et al. 2022; Pfurtscheller 1997; Pfurtscheller and Silva 1999; Doppelmayr et al. 2005; Caravaglios et al. 2015). Researchers are widely aware of the co-existence of these two brain response patterns and their respective functional and cognitive associations, but the mechanistic relationships between the two remain unclear. Based on their robust coexistence reported in the literature, we hypothesized that they are generated by the same underlying system which will be demonstrated in the present study. To this end, this study mainly comprises: (1) presenting the coherence of the neural activities across multiple levels and aspects (spikes, LFP, EEG) in different species (human and monkey), and (2) showing that a generic dynamic model of neuronal circuits can account for the key multilevel and multifaceted features and their inter-relationships. Our analyses showed that several aspects of data feature at multiple levels observed from the empirical data can be qualitatively reproduced in the simple neuronal network models constrained by generic E–I balance operating around critical states.

## Results

### Consistent neural response activity patterns across human EEG and monkey LFP

We presented neural response patterns measured by human EEG and monkey LFP signals to descriptively demonstrate the consistency in several aspects of unique features across species at multiple levels. Here, multiple levels refer to the different levels of organization in the neural system from which neural signals can be measured, e.g., single neurons, collective neural populations, neural functional systems, major cerebral lobes. In doing so, our aim is not to quantitatively and systematically prove the consistency at a fine-grained level such as the precise timing, detailed waveshapes of the signals, neuroanatomical sources or types of neurons generating the compared signals, and specific cognitive processes involved. Rather, the aim is to demonstrate the existence of key non-trivial features across levels and facets and their consistency in different species, such as coexistence of oscillatory dynamics and evoked transient response activities, and coarse-grained structure of response waveshapes. The data were from tasks that present stimulus and generate response, but the specificity of the tasks was not particularly relevant here. Despite being coarse-grained, these cross-species consistency and cross-level and -facet coherency are sufficient to demonstrate non-triviality and place strong constraints to the model.

**Fig. 1:**
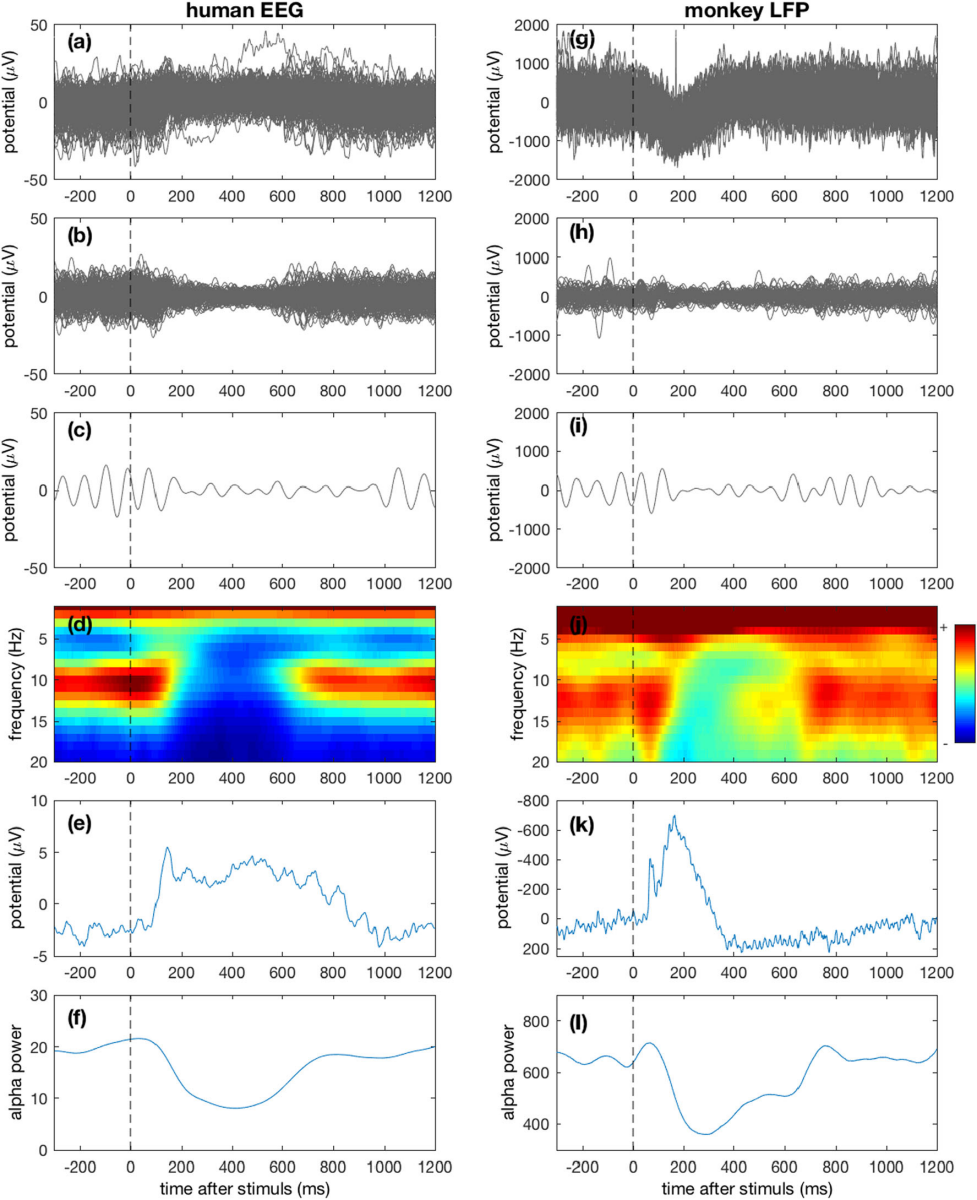
Key patterns of neural response activity from human scalp EEG (left column) and monkey LFP (right column). **a** Superimposed single trials of EEG data synchronized to stimulus onset from the occipital electrode PO9. **b** Same as (**a**) but band-pass filtered at 6–15 Hz to better visualize alpha waves. **c** An exemplified single trial from (**b**) showing clear post-stimulus alpha power suppression. **d** Time–frequency representation of single trial data in (**a**), averaged from all trials. **e** Average waveform of the single trials in (**a**), i.e., the event-related potential (ERP). **f** Average of the oscillation power in alpha band (8–12 Hz). **g**–**i** Same as (**a**–**e**) but for the LFP data from monkey. In (**l**), the curve was averaged from 10 to 15 Hz based on the alpha band observed in (**j**)

Figure 1 (left column) shows the key dynamic features of neural response activities measured from human scalp EEG. The time zero refers to the time point when the visual stimulus was presented. The superimposed single trials shown in Fig. 1a display a large amount of fluctuation reflecting the strong ongoing dynamic activity. Nonetheless, a systematic change of ongoing activity by stimuli in baseline shifting and oscillation suppression is clearly visible (Fig. 1a). The change of oscillation power can be more clearly seen in the band-pass filtered data. Figure 1b shows the same single trials of EEG data filtered within the bandwidth of 6–15 Hz to better show the dynamic change within alpha oscillation band (8–12 Hz) as a unique aspect of brain response. The alpha power is significantly suppressed during stimulus processing, and is restored after about 600 ms. This is a phenomenon commonly referred to as event-related desynchronization (ERD) which is associated with brain cognition and function [50–52). Figure 1c exemplifies the pattern of alpha suppression by a representative single trial EEG from Fig. 1b. To further present the temporal dynamic of alpha oscillation, we applied time–frequency wavelet analysis to the original single trial EEG data. The time–frequency representation averaged across trials reveals the detailed dynamic evolution of oscillations (Fig. 1d), from which we can see the change of alpha power (the dominant band around 10 Hz) along the time axis covering the stimulus processing. The alpha power is significantly decreased in the first few hundreds of milliseconds after stimulus onset (200 ms to 600 ms). Figure 1e and Fig. 1f show the average ERP response activity and the temporal pattern of alpha power (averaged within 8–12 Hz). The co-varying patterns of the temporal dynamics of ERP and ERD (alpha power) suggests a close relationship between these two different dynamic response activities.

To show the common features of the brain response dynamics between human and monkey, in Fig. 1, right column, we presented the LFP activity from the monkey’s lateral intraparietal cortex during the brain responses to the stimuli. We showed the results of monkey LFP for various aspects in the same order as presented in human EEG (Fig. 1, left column), i.e., single trials, filtered single trials, an exemplified trial, time–frequency representation, averaged ERP response activity and alpha power.

Several unique characteristics consistently exist in the neural activities from human EEG (Fig. 1, left column) and monkey LFP (Fig. 1, right column), which are summarized below.

1. Both show visually detectable, systematic response activities related to stimulus processing (Fig. 1a, e vs. Fig. 1g, k), within the first few hundreds of milliseconds after stimulus onset. Note that the two data types show different polarities because of the different depth of recording sites (Buzsáki et al. 2012).
2. Both show similar patterns of alpha suppression (Fig. 1b, c, d, f vs. Figure 1h, i, j, l). Notably, the distributions of alpha bands are slightly different: in human EEG the alpha oscillation occupies 8–13 Hz (Fig. 1d) and in monkey LFP alpha occupies 10–15 Hz (Fig. 1j).
3. The temporal patterns (including starting and ending time) of alpha suppression for human and monkey are similar (Fig. 1f vs. Figure 1l). Both are from around 200 ms to around 600 ms.
4. Both show similar waveform patterns of trial-average response activities (ERP, Fig. 1e vs. Figure 1k): a sharp peak followed by a wide hump. For human EEG data, the peaks appear later and prolonged. This is likely due to the difference of cognition complexity between the two tasks where the one for human may involve larger scale cortical networks.

The above comparison between human EEG and monkey LFP suggests a fundamental and generic organization of neural dynamic system underlying the brain’s ongoing and response activities, which should be further rooted in the basic neuronal firing activities. To demonstrate the link between collective activity and neural firing we further compared the LFP with multiunit neuronal spike activities both recorded from the same monkey subjects.

### Relationship between neuronal spikes and LFP in monkey’s cortex

**Fig. 2:**
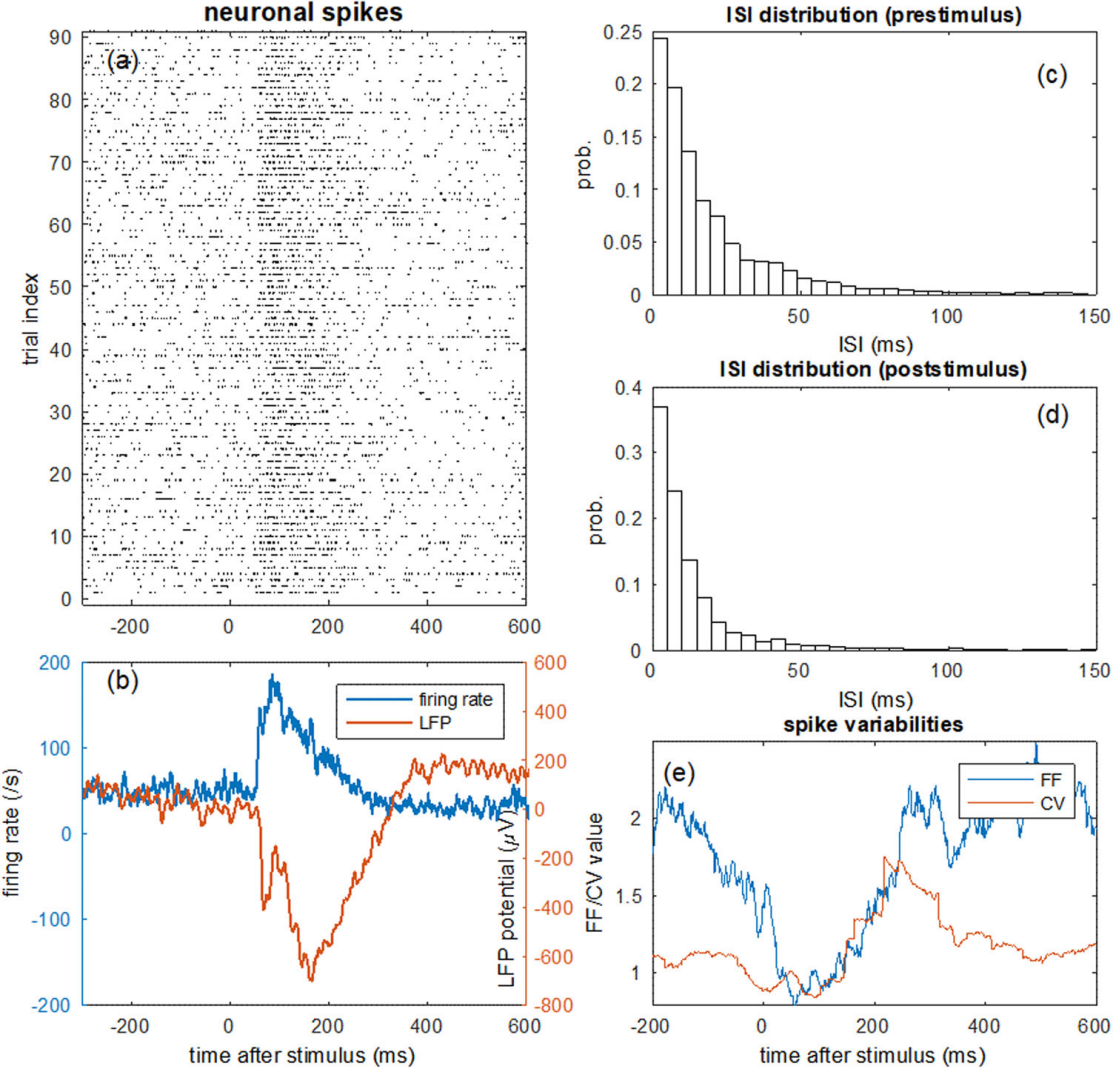
Multilevel and multifaceted neural response pattern in monkey’s brain. **a** Single trial neuronal spikes*.* **b** The estimated temporal course of firing rate of spikes from the trials (calculated in a moving window of 5 ms) in comparison with LFP-ERP averaged over LFP trials. **c** Distribution of pre-stimulus inter-spike interval (ISI) from − 1000 ms to 0 ms. **d** Distribution of post-stimulus ISI (0–400 ms). **e** Characteristics of spike variability calculated by CV and FF parameters with a sliding time window of 100 ms

The simultaneous recording of LFP and multiunit neuronal spike events allows us to examine the relationship between the spike activity and the collective activity pattern in LFP. The single trials of spike activity are plotted in Fig. 2a. It is visually apparent that the firing rate increased in response to the stimulus input, which demonstrates that the neurons were activated by the stimulation, and supports that firing rate carries information (Si and Sun 2021). The firing rate increasement is clearly visible in the firing rate curve calculated over the experimental trials shown in Fig. 2b (blue curve).

For comparison, we also plotted the time course of the average LFP across trials with respect to stimulus onset (Fig. 2b red curve), similar to ERP from human EEG. As shown, the response pattern of spike activity (in terms of firing rate) and the ERP from LFP (called LFP-ERP) are temporally closely associated with each other, as shown in the alignment of the starting points and covariation of the entire time course. Both firing rate and LFP-ERP curves resemble a typical ERP waveform from human brain corresponding to P1 and P3 components (Fig. 1). The temporal resemblance of firing rate and LFP-ERP provides concrete evidence on the common origin of the two signals from different levels of the underling neural circuits.

In neural coding, information may not be merely coded by neuronal firing rate (Kumar et al. 2010). It has been proposed that neural population maximizes the firing variability in resting state to best prepare for environmental uncertainty while reducing the variability in responding to the incoming signal (Friston et al. 2012; Cocchi et al. 2017). Therefore, in addition to firing rate pattern, we also examined other high order statistic features of spike activity in the aspect of variability, quantified by the coefficient of variance CV and the Fano factor FF described in the Method. The distributions of inter-spike interval (ISI) of the multiunit activities are shown in Fig. 2c, d for resting and processing states, which show that ISI is clearly reduced in the processing state. The time courses of CV and FF are shown in Fig. 2e. The CV value increased during stimulus processing whereas FF shows a clear reduction during response, indicating that the firing time of neurons becomes more irregular during the firing burst, but the rate is more reliable across trials.

These results from the empirical data clearly show close associations amongst neuronal firing rate and variability, ongoing oscillation patterns in LFP and EEG, the trial-averaged ERP and ERD (alpha suppression), and a high degree of consistency in these multifaceted dynamic features across human and monkey. The next question we want to address in this work is whether this high degree of coherency can be explained by the dynamics of a generic neural circuit network. To address this question, we studied a generic computational model of neural circuits based on the E–I balance neuronal network model described in the Method section.

### ***Unified explanation of neural activity in spikes, ERD and ERP based on a generic ***E–I*** balanced neuronal network***

We built the E–I balanced neuronal network model consistent with our previous work (Yang et al. 2017; Liang and Zhou 2022; Liang et al. 2020) (see Method) to understand the relationship between the microscopic and macroscopic neural activities revealed in the empirical data summarized above. We created a relatively dense network capturing the basic biological ingredients of dense connection among excitatory and inhibitory neurons in local cortical circuits, whose behavior is not a simple asynchronous state studied previously in large and sparse network (Vreeswijk and Sompolinsky 1996; Vogels and Abbott 2005, 2009), but jointly exhibits stochastic oscillations and critical avalanches in the presence of background input in a wide parameter region of coupling strength (Wang et al. 2011; Wang et al. 2016), consistent with experimental observations of oscillations and avalanches (Gireesh and Plenz 2008).

The dynamics of the network model changes with the coupling strengths $\Delta {g}_{ex}$ and $\Delta {g}_{inh}$. The network tends to be inactive if inhibitory couplings are too strong, but fires rather regularly with a high frequency if the excitatory couplings are too strong. In a broad intermediate range of the coupling strengths, the network self-organizes into effectively balanced states where neuronal spikes are irregular and weakly correlated. The critical state with low-rate irregular spiking can be obtained between the inactive and the regular states as the synchrony between neuronal spikes increases. Due to the finite size effect, the network in the critical couplings regime may also be occasionally initialized into a state exhibiting irregular activity with low synchrony and high firing rate that does not display power-law feature. We excluded these simulation realizations of low correlation and only considered the states exhibiting criticality, which is robust to the system sizes and external input (Liang and Zhou 2022). For efficient of computation, we simulated a reasonably small network size of 500 neurons.

**Fig. 3:**
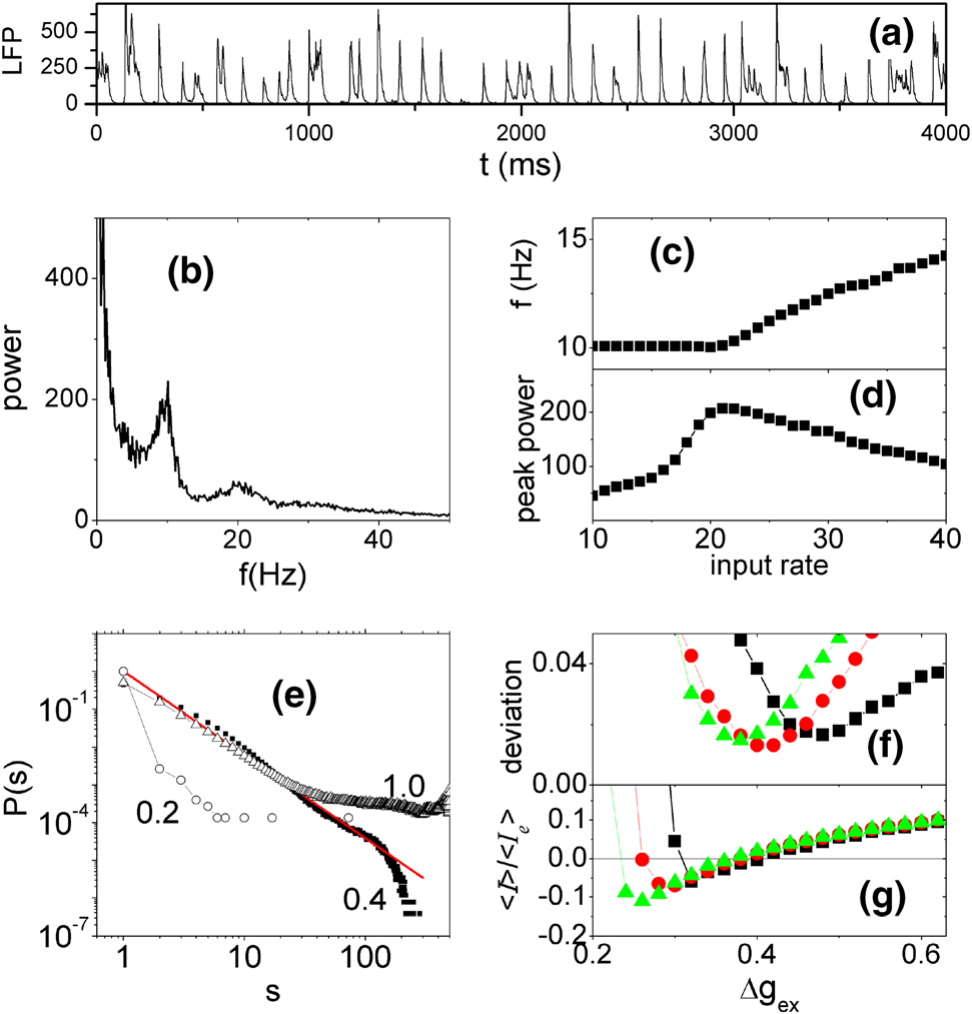
Dynamical patterns of E–I balanced network with background input at critical state. In **a**, **b**, **e** and **f**, the coupling parameters are $\Delta {g}_{Inh}=5.0, \Delta {g}_{ex}=0.4,$ and the background input rate is 20 spikes/second for each neuron. **a** LFP of the network. **b** Power spectrum of the LFP signal in (**a**). **c**, **d** Changes of peak frequency and peak height of the power spectrum as a function of the background input rate. **e** Distribution of the size of neuronal avalanches at $\Delta {g}_{ex}=0.2, 0.4, 1.0$. The red line is a power-law function fitted to the distribution at $\Delta {g}_{ex}=0.4$. The K-S test *p*-value is 0.37. **f** Deviation of avalanche size distribution from the best-fitting power-law function versus the excitatory coupling strength. To discard the cutoff, the distribution curve above $5\times {10}^{-5}$ was used in the power-law fitting. **g** The balance of excitatory and inhibitory couplings represented by the ratio of the mean synaptic current of a neuron to its mean excitatory synaptic current. The currents are averaged over a time window of 2000 ms and the ratio is averaged over all neurons in the network. The background input rates are 20 Hz, 30 Hz, and 40 Hz for results represented by black squares, red circles, and green triangles, respectively

The behavior patterns of the neural network model with 500 neurons under background input are summarized in Fig. 3. The LFP signal represented by synaptic current $\left|{I}_{Ex}\right|+\left|{I}_{Inh}\right|$ resembles the 10 Hz oscillatory pattern (Fig. 3a) in empirical data, though the latter is smoother, likely due to larger neural population. The power spectrum of the LFP signal is presented in Fig. 3b. The alpha stochastic oscillation persists across a broad range of input rate under random weak inputs, as shown by the peak frequency and power as functions of the background input rate in Fig. 3c and d. The alpha oscillation exists under the range of 10–40 Hz background input rate for each neuron. Under weaker input (< 10 Hz), the network cannot be activated to maintain ongoing activity and keeps silent. Stronger inputs (> 40 Hz) induce fluctuating activities with higher and broader frequency band. The change of frequency in the gamma band with external input was also observed previously in the model (Liang and Zhou 2022).

This model brings collective network oscillation and irregular neuronal spiking into co-existence, supported by the critical state of the network due to E–I balance. Our recent theoretical study using mean field model showed that such co-existence is related to a Hopf bifurcation from fixed point to oscillatory state in the mean field (Liang et al. 2020). The power-law distribution of neuronal avalanche size is the fingerprint of the critical state (Gireesh and Plenz 2008). For fixed inhibitory coupling $\Delta {g}_{Inh}=5.0,$ the avalanche size distributions in Fig. 3e for various values of excitatory coupling strength $\Delta {g}_{ex}=0.3, 0.4, 1.0$ show a transition from subcritical to supercritical firing. We recorded the size of each avalanche (52,000 in total) in the network with ∆g_ex_ = 0.4 under the stimulus of 30 Hz. The distribution of avalanche sizes was fitted into doubly truncated discrete power law using the method of maximum-likelihood estimation to find the ranges that pass the truncation-based Kolmogorov–Smirnov (K–S) statistics test (with p values larger than 0.2). The fit range satisfying *p* > 0.2 was automatically detected by the NCC toolbox (Marshall et al. 2016) as between 9 and 253. With this fit range, the K-S test *p*-value is 0.37. Therefore, the truncated power law is an acceptable fit of the avalanche size distribution. To further quantify the transition, we calculated the deviation of the distribution from the best-fitting power-law function (Wang et al. 2011, 2016) with different excitatory coupling strength $\Delta {g}_{ex}$, under different background input rates. The deviation is minimized in the critical state. The curve of the deviation shows that the critical state appears at around $\Delta {g}_{ex}=0.4$ for a range of background input rates that generates alpha-band stochastic oscillations (Fig. 3f).

We also investigated what role the balance between excitatory and inhibitory currents in the network plays in the co-existence of the multilevel activity. The network is silent when the excitatory coupling strength $\Delta {g}_{ex}<0.2$. As $\Delta {g}_{ex}$ increases, both excitatory and inhibitory neurons are activated. Inhibition first dominates the network and is later counter-balanced by excitatory synapses, approaching zero mean (balanced) synaptic current at around $\Delta {g}_{ex}=0.4$ (Fig. 3g), which matches the minima of the deviation of avalanche size distribution from power-law function (Fig. 3f). At the E–I balance, the dynamics of neurons are driven by fluctuations and self-organize into critical spiking avalanches and generate collective stochastic oscillations in the alpha band due to an accumulation-and-release process (Wang et al. 2016), theoretically associated to Hopf bifurcation in the corresponding mean field model (Liang and Zhou 2022; Liang et al. 2020). The peak frequency and power of the alpha band vary as functions of the background input strength (Fig. 3c, d).

These results show that a generic neuronal network capturing the basic biological features of local densely connected neural circuit and conductance-based excitatory and inhibitory neurons with E–I balance can simultaneously produce the multilevel ongoing neural dynamics of irregular firing, critical avalanches and alpha band stochastic oscillations as typically observed in experimental data.

**Fig. 4:**
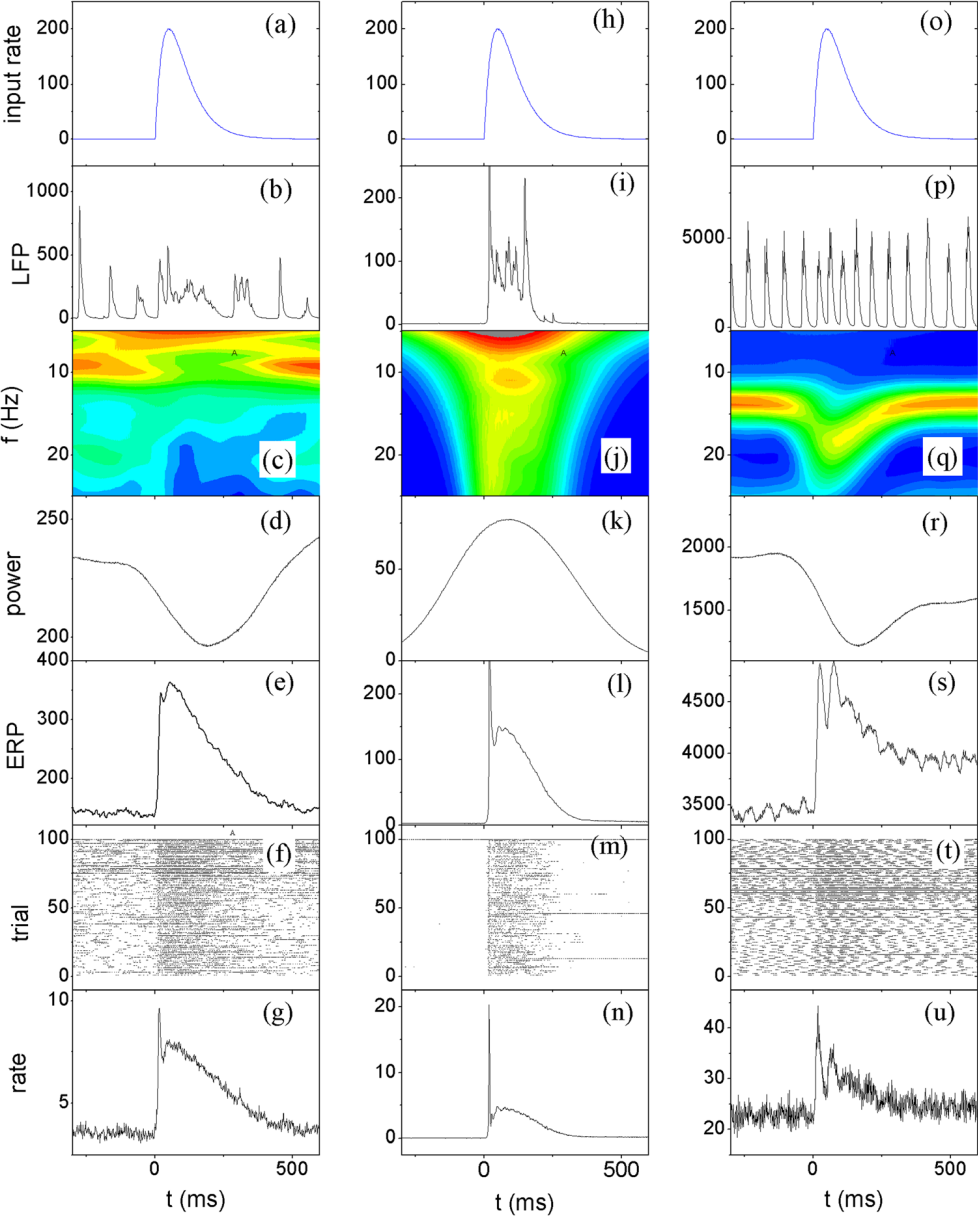
Response of the neuronal network to external input under different ongoing states. Left column **a**–**g**: critical state with $\Delta {g}_{ex}=0.4$ and $\Delta {g}_{inh}=5.0$; Middle column **h**–**n**: subcritical state with $\Delta {g}_{ex}=0.25$ and $\Delta {g}_{inh}=5.0$; Right column **o**–**u**: supercritical state with $\Delta {g}_{ex}=1.0$ and $\Delta {g}_{inh}=5.0$. **a**, **h**, **o**: Spike rates of the external input signal. **b**, **i**, **p** an exemplified single trial of LFP signal of the network. **c**, **j**, **q** Time frequency representation of the LFP signal, averaged over 100 trials. Warmer color represents higher power. **d**, **k**, **r**: Time evolution of alpha power (8–12 Hz). **e**, **l**, **s** LFP-ERP waveform. **f**, **m**, **t** Spike raster of 20 randomly selected neurons in the network. **g**, **n**, **u** Firing rate of the network. The alpha power, LFP-ERP and firing rate were obtained by averaging over 1000 trials

Next, we studied the response of the network at the critical ongoing state to model the generation of ERP and ERD in association with the changes in spiking, as observed in the experiments. The external input given to the critical-state network was simulated according to an alpha-function^1^$r = ate^{{ - \frac{t}{\tau }}}$ for the spike rate as illustrated in Fig. 4a, starting from t = 0. The maximal rate reached at $t = \tau$ is $r_{m} = a\tau /e$. We set $\tau$ = 50 ms, and $r_{m} = 200$ Hz.

The alpha oscillation in LFP signal is suppressed due to the external input (Fig. 4b) and is recovered later when the input vanishes. The alpha suppression is also clearly presented in the time frequency plot (Fig. 4c) and in the time course of alpha power in LFP (Fig. 4d). The LFP-ERP averaged across trials (Fig. 4e) shows a small sharp peak shortly after stimulus onset, followed by another broader peak, qualitatively similar to the ERP from monkey LFP (Fig. 1).

The changes in the oscillation patterns and ERP are rooted in the neuronal spike activities in the network. The spike raster (100 trials) shows that the input stimulus increases the neuronal firing rate (Fig. 4f), which is expected to reduce alpha power according to the relationship shown in Fig. 3. To mimic the experimental measurement of multiunit spikes of the neurons close to the electrode, here 20 randomly selected neurons were used to simulate the multiunit spike activity. The average spike rate (for each neuron) across trials (Fig. 4g) also exhibits a sharp peak followed by a broad one, very similar to the ERP pattern. The ratio of the peak rate of the response to the ongoing activity is about 3, which is also similar to the monkey firing data (Fig. 3). The results above show that when the ongoing dynamics of network operates at the E–I balanced region (at critical states), the response to external stimuli can simultaneous produce the co-varying patterns of firing rate, ERP and ERD as consistently observed in human EEG and monkey LFP. The response features presented above for a given stimulus duration and strength are robust against the duration and strength of the stimuli, see S1 Fig in Supporting Information.

The behavior of the critical state neural network model persists in large modular networks built by coupling the small dense modules through sparser inter-modular connections. Starting from random initial conditions, the activities from other modules act as the background input to each module, thus the modular network can self-sustain ongoing activity without the need of adding additional background activity. In the modular network, each module is also in the critical state. The avalanche size distribution of modules and the response to external stimulus are shown in S2 Fig in Supporting Information, which are also qualitatively similar to experimental observations.

**Fig. 5:**
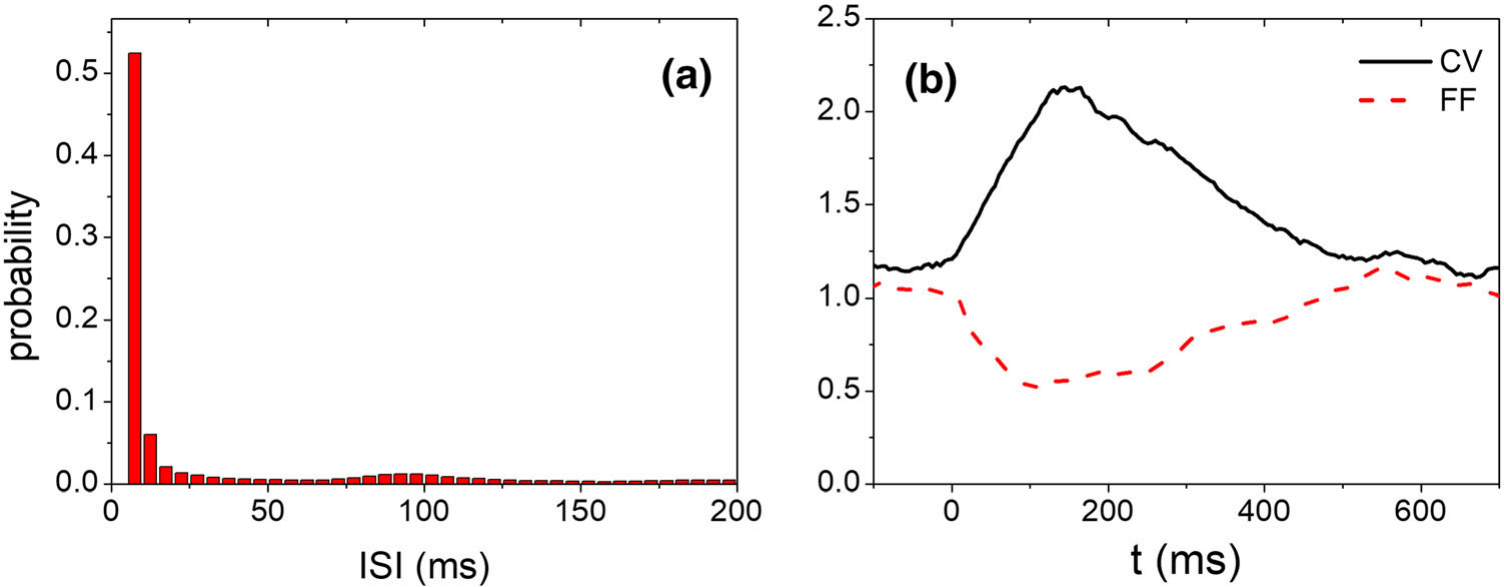
Variability of neuronal spiking from the network in critical states. **a** Distribution of ISI of neurons with background activity. **b** Temporal variability (CV) and cross-trial variability (FF) of spikes, calculated in a moving window of 100 ms. External stimulus is added at t = 0. The network parameters and the input rate are the same as the Fig. 4a–g

Different from the oscillatory patterns in the collective behavior of the circuits, the single neurons fire spikes irregularly, as revealed by a broad distribution of inter-spike intervals with a slightly preferred spike interval around 100 ms (Fig. 5a) due to the modulation from the collective alpha oscillations. The value of CV (1.28) from the ongoing dynamics of the model is at the same level of what was commonly found experimentally (CV ~ 1–1.5, according to Shadlen and Newsome (1998)), also quite close to the CV values of pre-stimulus stage of the monkey multiunit data in Fig. 2e. Along with the change of the firing rate, the temporal variability and the trial-to-trial variability of the firing activity are also changed (Fig. 5b), which is consistent with the results from monkey cortex (Fig. 2e).

We emphasized that the simultaneous reproduction of the multilevel and multifaceted key features in the experiments by the same model only occurs when the network is operating around critical states. In sub-critical states, the network with weak background input has very sparse activation (Fig. 4m) and the avalanche size distribution strongly deviates from a power-law function (Fig. 3e). Before the onset of the stimulus, the background input could not generate ongoing oscillations in the single trial LFP (Fig. 4i), and the stimulus generates irregular LFP with a broad range of frequencies (Fig. 4j) due to the transient burst of firing of the neurons (Fig. 4m). The change of alpha power, waveform of ERP and average firing rates are substantially different from experimental data, though ERP and firing rate curve still display a sharp pulse followed by a lower peak. At super-critical states (Fig. 4o-u), the ongoing activity and the response of neural networks also differ from experimental data. The avalanche size distribution shows higher probability of large avalanches (Fig. 3e). The ongoing activity displays very regular oscillations (Fig. 4p). The oscillation is so regular that there are multiple peaks on the power spectrum. Such pattern is rarely seen in experimental data. The stimulus input shifts the central frequency of the ongoing oscillation instead of suppressing its power (Fig. 4q).

## Discussion

In this paper, we illustrated several key features in the ongoing and response activity of multilevel neural system based on human EEG and monkey LFP data from typical task experiments, highlighted the cross-level coherency and cross-species consistency, and presented a computational model of neuronal network that reproduces the temporally matched multilevel and multifaceted features in the experimental data, particularly, in the spiking dynamics, ERP and ERD. This work was motivated by the limitations in studies that attempted to characterize neural activity pattern at a single level and understand the generative model thereof. Of note, this work aims to demonstrate generic principles in neural systems that are not restricted to neural functional or anatomical specificities. As such, the data sources were selected to demonstrate the existence of multiple classic neural features, not to represent functional specificity from special anatomical locations (the specific location of the data source is thus not highly relevant to the theme). We argue that the multilevel coherency of neural activities, including spontaneous neural dynamics and response patterns in neuronal spiking, avalanches, ongoing oscillations, ERD and ERP, place strong constraints on the mechanisms and principles in a generative model. As such, a simplistic model that follows generic principles and simple parameters being able to simultaneously reproduce key neural activity features at multiple levels of organization (e.g., single neuron, local cortical areas) including multiple aspects (e.g., spiking rate, variability, irregularity, transient response pattern, oscillations) is by no means trivial, but rather demonstrates strong merit by providing possibility of understanding complex brain dynamics through unified principles. On the other hand, given the actual complex details in empirical data and the aim of addressing the cross-level and cross-species consistency, the scope of our modelling is positioned to only reproduce the existence of key data features at different levels and in major aspects (thus is a qualitative reproduction), not to achieve fine-grained match in all subtle details. Adding the present finding to previous ones (Yang et al. 2017; Liang and Zhou 2022; Liang et al. 2020), the multilevel and multifaceted neural features that an E–I balance model can reproduce include irregular firing of individual neurons, critical avalanches, complex collective fluctuations with pronounced oscillations (alpha waves) in spontaneous states and stimulus-elicited changes in neuronal firing rate, ongoing oscillations (ERD/S), and basic waveform pattern of trial-averaged ERPs. These results provide potential dynamical mechanism underlying the organization of multilevel complex neural dynamics in the brain.

### Nontriviality of the multilevel and multifaceted coherency and cross-species consistency in neural activities

The observed multilevel and multifaceted neural activity features reflect a unified dynamic system organization of the circuits, but not a piecemeal set of multiple features. Our characterization showed a high degree of coherency and consistency in the data that are important for modelling. To summarize, the first significant feature is the two-thread response at the collective activities that are consistent in human EEG and monkey LFP data: generation of a transient dynamic response (ERP) and suppression of ongoing oscillation (Alpha). These two threads precisely match in timing (Fig. 1), suggesting that they are from the same or a highly integrated dynamics system. This feature places a strong constraint to the underlying model: (1) the model has to possess a self-organized oscillation that displays a biologically plausible frequency (around 10 Hz), (2) the ongoing oscillation should be functionally relevant to the system’s response to external inputs, (3) the response in the spontaneous oscillation is suppression but not enhancement, (4) the transient dynamic response waveform should follow the overall structure in the empirical data.

The second significant feature is the matchness of the temporal dynamic pattern of neural response manifested in neuronal firing and LFP (Fig. 2). This provides solid evidence that the macroscopic activity patterns could be largely determined by the elementary activity of neuronal firing, which also suggests the plausibility of simulating the observed multilevel features simultaneously based on a generative model that is constructed on single-neuron dynamics, rather than relying on a phenomenological model to explain macroscopic data. To explain macroscopic patterns in a bottom-up approach starting from the elementary level of organization of neurons, it is crucially important to capture the essential dynamic principles and biological reality in the generative model. These dynamic principles will be discussed below.

### Neural dynamics accounts for the multilevel consistency based on generic E–I balanced model and criticality

The multilevel complexity and consistency inspired us to seek a generic explanation incorporating key neurobiological factors and dynamical principles and architectures. One important factor is E–I balance (Shu et al. 2003; Haider et al. 2006). Theoretically, sparse network of excitatory and inhibitory neurons with E–I balance can self-organize asynchronous and irregular activity (Vreeswijk and Sompolinsky 1996; Vogels and Abbott 2005, 2009), which provides a mechanism for the irregularity of neuronal firings. However, completely asynchronous state cannot produce clustered firing (avalanches) and collective oscillations in the network, thus is not consistent with cortical dynamics in the macroscopic level.

Our recent work has addressed this gap by a systematic study of similar E–I balanced neuronal networks simulated in this work (Yang et al. 2017; Liang and Zhou 2022; Liang et al. 2020). We showed that in E–I network with dense enough connections, there is a dynamical regime unifying irregular spiking, neural avalanche and collective oscillation (Yang et al. 2017), corresponding to transition in the mean field model from fixed point (asynchronous state) to oscillatory state through a Hopf bifurcation (Liang and Zhou 2022; Liang et al. 2020). When the system is operating around the self-organized critical regime where activation of one neuron can induce critical branching of activation to other neurons in the circuit (statistically neither decaying nor growing) due to E–I balance, the accumulation of neural activation from weak background input is sufficient to cause avalanches of various sizes. However, the E–I current has a loose balance (Liang and Zhou 2022), which is temporally broken down to generate oscillations due to E–I feedback loop, which can be in the alpha band, with oscillation frequency depending on external inputs (Fig. 3c). In the presence of external stimulus, more neurons can be transiently activated, the slow accumulation and large release process of avalanches are replaced by frequent and local avalanches, leading to suppression of the original alpha oscillations and increased firing rate, thus explaining the effect of Alpha suppression more broadly known as ERD. In sum, the simple neural network operating around E–I balance with critical ongoing dynamics is sufficient to reproduce key aspects of activity pattern observed in the real data of human EEG and monkey LFP in the presence of external stimulations: (1) Coexistence of irregular neuronal spikes and oscillatory patterns in the activity of neuron ensemble. (2) Simultaneous manifestation of neural response in evoked transient activity (ERP), suppressed oscillation, increased firing rate, change of the temporal and cross-trial variability of neuronal firing. (3) Matchness in the timing of the multilevel responses. These results are consistent with our recent study focusing on the reduction of trial-to-trial variability in the neural spiking and LFP, increased frequency of gamma oscillations and preserved dynamical criticality by external stimulus in the E–I balanced model operating around critical state (Liang and Zhou 2022). There it was also confirmed that these multiple features of reduced variability, increased gamma frequency and preserved criticality widely observed in different experiments cannot simultaneously occur in non-critical dynamical states. Here in this work, we extended the scope to reveal the consistency in the reduced variability with enhanced firing rate, ERP and ERD generated by external stimulus, which is also exclusively observed in the critical state of the ongoing dynamics.

Such dynamical properties simultaneously emerge without sophisticated connection structure, special neuronal dynamics, and the need of fine-tuning of parameters, which serves as fundamental dynamical principle underlying complex spontaneous and response neural dynamics. However, we reiterate the point that such simple network only reproduces the existence of key phenomena observed in multilevel empirical data that has strongly upheld the non-triviality of the generative model, but not the detailed quantitative features in the data that are produced by specific biological features and need hard-code tuning in the model. For example, the variability (CV) of the model is slightly larger than that observed in LFP; the broad peak in ERP and firing rate from monkey LFP do not exactly match in timing; the frequencies of Alpha oscillation in human EEG and monkey LFP do not match. Future studies can use such E–I balanced network as a core model with additional biological details to study more specific information processing associated to the multiple neural response features to external stimulus, such as learning and memory (Li et al. 2021b; Li et al. 2022).

## Concluding remarks

Our analyses provided an integrative understanding of the multilevel and multifaceted neural activity features in resting state and response to external input based on a generic E–I balanced neuronal network, extending previous reconciliation of irregular spiking, critical avalanches and collective oscillations in spontaneous state and the stimulation-reduced variability, change of frequency and preservation of criticality to further embrace the reconciliation of firing rate, ERP and ERD. This modelling approach treats the entirety of the observed neural activity signals, including the ongoing non-task signals as an integral and functional part from an integrated complex neural dynamical system, and attempts to offer a possibility of understanding complex neural data by incorporating both biological reality and generic principles. The demonstration in this work is only at the stage of presenting the general conceptual framework. The validation of this approach should be further supported by future studies that focused on addressing real and subtle phenomenon or effects in a quantitatively precise manner. Such a model that can simultaneously reconcile multiple level and multifaceted features of neural spontaneous and evoked dynamics can serve a good computation and modeling template for studying how these dynamics feature impact on information processing in normal and abnormal neural circuits.

## Method

### Empirical neural data

For the present research purpose, we included scalp EEG signal from human, LFP and neuronal firing activities from rhesus monkey. The data were from different independent experiments devoted to address different neurocognitive research questions for which specific experimental setups and manipulations were employed. However, with a focused interest in fundamental questions about the genesis of the multilevel and multifaceted neural features, the current work will only focus on the pre-stimulus and post-stimulus segments of neural signals. Therefore, the high-level experiment manipulations are out of the current scope, thus only basic and relevant information of experiment setups is described below.

The data analyses were focused on the changes in the dynamic pattern of neural activities in different levels with respect to the stimulation. Throughout the article the time point zero refers to the presentation time of stimulus, i.e., stimulus onset. Segments of neural activity, be it neuronal spikes (and its rates), LFP or EEG, from pre-stimulus to post-stimulus time window will be presented and analyzed, in both single trial and average levels. The advantage of using neural activity segments encompassing stimulus onset is that both resting state, response activity and recovering features can be examined.

For the presentation of the time–frequency pattern of temporal neural data from experiments and model, wavelet analysis was applied on the single trial segments with Morlet wavelet basis. Average time–frequency pattern was then obtained by averaging the modulus of complex wavelet parameters of all single trials to show the average pattern of brain response in time–frequency representation.

#### Human EEG data

The human EEG data was taken from Verleger et al. (2014) investigating the relationship between ERP waveform pattern and frequency of stimulus types in the stimulus sequence. Twenty participants conducted an ‘oddball’ task in which a letter (X or U) was presented repeatedly in random order while the EEG data was recorded from the scalp. Participants were required to respond to one of the letters by button-pressing, but only when a frame surrounding the letter was shown. One of the letters was frequent (80% of trials), the other was rare (20% of trials). The continuous EEG data was segmented into single trials associated to each stimulus, i.e., letter X or U. More technical details of the experiment can be found in Verleger et al. (2014).

#### Monkey LFP and neural firing activity

The experiment on monkey was designed to study the role of cortical area lateral intraparietal (LIP) in the generation of express saccade (Chen et al. 2013). Two rhesus monkeys were trained to perform saccadic eye movement towards the dot stimulus which was presented after the fixation cross with a certain displacement. The LFP activity signal was measured using a single electrode at LIP during the task processing and the multiunit spiking events near it were recorded. The continuous LFP data was segmented into single trials associated with each stimulus. More technical details of the experiment can be found in Chen et al. (2013).

### Dynamic model of neuronal network

#### Model and parameters

The maintenance of E–I balance in biological neural network has been demonstrated experimentally (Shu et al. 2003; Renart, et al. 2010). To mimic the micromodules of local cortical networks, we analyzed an isolated, small, and dense random network of excitatory and inhibitory neurons with weak background input. The network is composed of *N* = 500 leaky integrate-and-fire neurons (80% of them are excitatory) coupled with a connection density *p* = 0.16. The main results reported here are robust when the network is scaled to a larger size with properly adjusted coupling density to keep networks at E–I balanced and critical states. The dynamics of a single neuron (Vreeswijk and Sompolinsky 1996; Vogels and Abbott 2005) is formulated as follows:

1 $$ \begin{array}{*{20}c} {\tau \frac{dV}{{dt}} = \left( {V_{rest} - V} \right) + g_{ex} \left( {E_{ex} - V} \right) + g_{inh} \left( {E_{inh} - V} \right).} \\ \end{array} $$

The membrane potential of a neuron is denoted by $V$, with a time constant $\tau$ and a resting membrane potential $V_{rest}$. The neuron fires a spike when $V$ crosses a threshold (− 50 mV) and $V$ is reset to $V_{rest} =$  − 60 mV and fixed for a refractory period (5 ms). The neurons are coupled through conductance-based synapses where $E_{ex} = 0 mV$ and $E_{inh} = - 80 mV$ are reversal potentials for excitatory and inhibitory synapses, respectively. The spiking of excitatory (or inhibitory) neuron increases the synaptic conductance of postsynaptic targets by $\Delta g_{ex}$(or $\Delta g_{inh}$), which decays exponentially with time constant $\tau_{ex}$ (or $\tau_{inh}$). The biologically plausible values of parameters were taken from Vogels and Abbott (2005): $\tau =$ 20 ms, $\tau_{ex} =$ 5 ms, and $\tau_{inh} =$ 10 ms.

To mimic the background input to the local circuit from other circuits in the neighborhood, each neuron receives a random spike train generated from a Poisson process with a low rate of 20 spikes/second. External stimulus input is modelled by a Poisson process whose rate changes over time. The spike rate of the stimulus starting from *t* = 0 is an alpha function $r = ate^{{ - \frac{t}{\tau }}}$, with maximal rate $r_{m} = a\tau /e$ reached at $t = \tau$. Experiments have shown that rats process olfactory sensory signals in 200 ms (Uchida and Mainen 2003), and signal processing in human visual system can be finished in 150 ms (Thorpe et al. 1996). According to these experiments, we considered it reasonable to select the value of $\tau$ in the range (10, 100) ms. We set $\tau$ = 50 ms, and $r_{m} = 200$ spikes/second.

To compare the behavior of the model to the features in LFP from the experiment, we estimated the mean excitatory synaptic current ($I_{Ex}$) and inhibitory synaptic current ($I_{Inh}$) of neurons and used $\left| {I_{Ex} } \right| + \left| {I_{Inh} } \right|$ to approximate the LFP signal of the neural network model (Mazzoni et al. 2008). Although the mean synaptic current may not be a precise model of LFP due to layered organization of cortical neural circuits (P. beim Graben, S. Rodrigues 2013), it reflects the main features of collective oscillatory behavior in the network.

#### Avalanche size distribution and deviation from power-law fitting in the model

Around the critical states, weak input drives the network to fire spikes in an intermittent way. When a neuron is activated by input spikes, the signal sent from the neuron to its network neighbors may activate those in the vicinity of firing threshold. The signal propagation in the network generates avalanches which stop randomly. The number of neurons firing spikes in an avalanche is defined as the size of the avalanche. In our simulation, a separate avalanche is defined in such a way that two avalanches are separated by one or more simulations steps during which no neuron in the network fires.

At critical state, the avalanche size distribution is a power-law function with an exponential cutoff due to the finite size of the network. The critical state can be identified by measuring the deviation of the avalanche size distribution from a power-law function. Only the left-hand side of the distribution function was used in the fitting. Using least square fitting, we obtained the mean square deviation of the data from the fitted curve. The critical state has the minimal value of the mean square deviation.

#### Variability measure for neuronal spikes

Temporal variability of neuronal spikes is measured by coefficient of variation (CV) calculated as the standard deviation of inter-spike interval (ISI) divided by the mean ISI (Shadlen and Newsome 1998). For a Poisson process, CV = 1. For a periodic process, CV = 0. Variability across trial is measured by Fano Factor (FF) calculated as the variance of spike number within a time window across trials divided by the mean number of spikes in this window (Britten et al. 1993). For a homogeneous (with a constant rate of events) Poisson process, FF = 1. For a periodic process, FF = 0.

### Supplementary Information

Below is the link to the electronic supplementary material.

Supplementary file1 (DOCX 179 KB)

## Data Availability

The datasets generated during and/or analysed during the current study are available from the corresponding author on reasonable request.

## Footnotes

1 The ‘alpha’ here refers to the shape of input signal described by the alpha function as formulated in the text. Note that this is different from the alpha oscillation mentioned throughout the article, which means a specific neural rhythmic activity with a frequency of about 10 Hz.
