# Supplementary material for: Multilevel and multifaceted brain response features in spiking, ERP and ERD: experimental observation and simultaneous generation in a neuronal network model with excitation–inhibition balance
Source: Cogn Neurodyn. 2022 Nov 23;17(6):1417–31. doi: 10.1007/s11571-022-09889-w (PMC10640466; doi:10.1007/s11571-022-09889-w)
Supplement: Supplementary file 1 — Supplementary file1 (DOCX 179 KB) [file 11571_2022_9889_MOESM1_ESM.docx]

**Supporting Information**

**S1 Appendix. The variance of the parameters in the input rate function**

The response behavior of the critical network is robust against the variance of parameters in the firing rate function. Experiments have shown that rats process olfactory sensory signals in 200 ms [1], and human visual system processing signals can be finished in 150 ms [2]. In our experimental results, the peak of LFP-ERP appears in several hundreds of milliseconds. According to these experiments, we think that the selection of the value of $\tau$ is reasonable in the region (10, 100) ms.

Under a wide parameter region of both the time scale and the strength of the external stimulus, the response of the critical network exhibits qualitatively similar ERP signal, see S1 Fig.

1. Uchida N, Mainen ZF. Speed and accuracy of olfactory discrimination in the rat. Nat Neurosci. 2003; 6(11): 1224. doi: 10.1038/nn1142.
2. Thorpe S, Fize D, Marlot C. Speed of processing in the human visual system. Nature. 1996; 381(6582): 520-522. doi: 10.1038/381520a0.


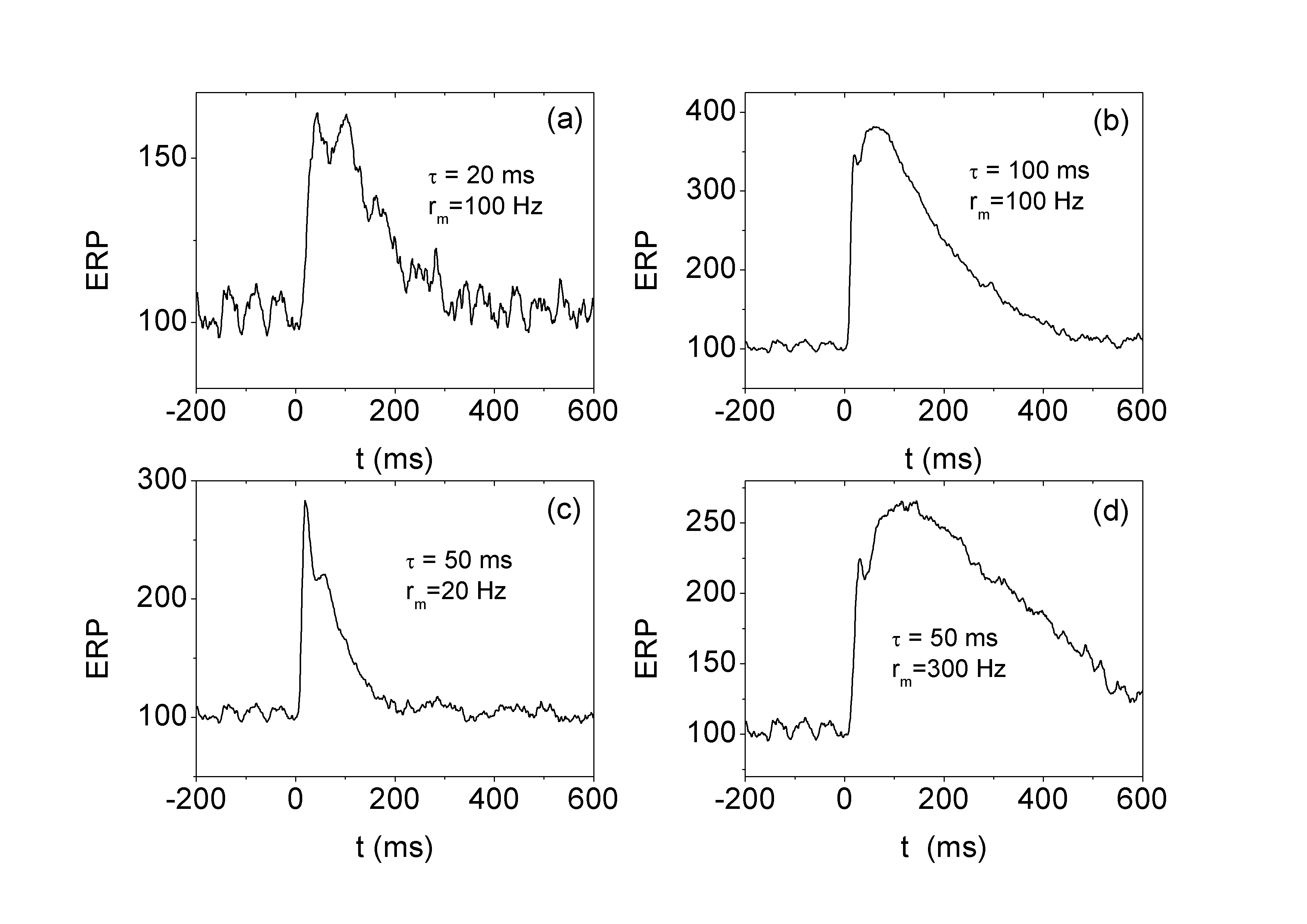


**S1 Fig**. **The variance of the parameters in the input rate function.** (a) Response to fast external stimulus. $\tau=20$ ms. The peak input rate is 100 spikes/second for each neuron. (b) Response to slow external stimulus. $\tau=100$ ms. The peak input rate is 100 spikes/second for each neuron. (c) Response to weak input. $\tau=50$ ms, the peak input rate is 20 spikes/second for each neuron. (d) Response to strong input. $\tau=50$ ms, the peak input rate is 300 spikes/second for each neuron.

**S2 Appendix. The Modular Network**

The behavior of critical state neural network model persists in large modular networks built by coupling the small dense modules through sparser inter-modular connections, which confirms the generality of results to larger scales. In the simulations we used 20 modules to build a modular network. We placed these modules on a 1D space in a consecutive way with the first and last module being further connected to form a ring (periodic boundary condition at module level). Within each module each pair of neurons are connected with the probability $p_{i}=0.16$, which is set to be the same as the connection density of an isolated small network. The connection probability between two neurons in module $i$ and module $j$ depends on the distance between the two modules, following exponential decay [1] as $p_{ij}=p_{1}e^{-\frac{\left| i-j \right|-1}{l}}$, where the coefficient $p_{1}$ is used to adjust the mean connection density. The mean inter-modular connection probability is $\bar{p}=\frac{1}{N_{m}}\sum_{i=1}^{N_{m}} \left( \frac{1}{N_{m}-1}\sum_{j=1,j\neq i}^{N_{m}} p_{ij} \right)$, which is set to be 0.0002. The parameter $l$ determines the locality of the inter-modular links and is set as $l=5$.

Starting from random initial conditions, the activity from other modules can act as the background input to each module, thus the modular network can self-sustain ongoing activity without the need of additional background activity. In the modular network, each module is still in the critical state. The power-law distribution of avalanche size of one module is shown in S2 Fig (a).

We simulated the response of the modular networks to an external input. In the simulation, no background stimulus was added. One of the 20 modules received an external stimulus added at $t=0$. LFP signal of the stimulated module was recorded. As shown in S2 Fig c,f, the ongoing activity of LFP exhibits an oscillation in the alpha band. When the stimulus is added at t=0, the activity of LFP transfers into fast fluctuations leading to suppression of the alpha oscillation which recovers afterwards.

The ERP signal was computed by averaging the LFP signal over 100 trials, and is shown in S2 Fig d. The ERP signal is very similar to that in the isolated module with a Poisson process background input (Fig. 4e). The firing rate of the stimulated module is shown in S2 Fig e, also similar to the isolated module (Fig 4g). We also showed the slow oscillations and its suppression by external stimulus using wavelet analysis in S2 Fig e. The power of the frequency band from 10 to 15 Hz is shown in S2 Fig f. These features of response obtained by simulations of the critical modular neural network agree with the key features observed in experimental results.

1. Markov, N. T., Misery, P., Falchier, A., Lamy, C., Vezoli, J., Quilodran, R., ... & Huissoud, C. (2010). Weight consistency specifies regularities of macaque cortical networks. Cerebral cortex, 21(6), 1254-1272.


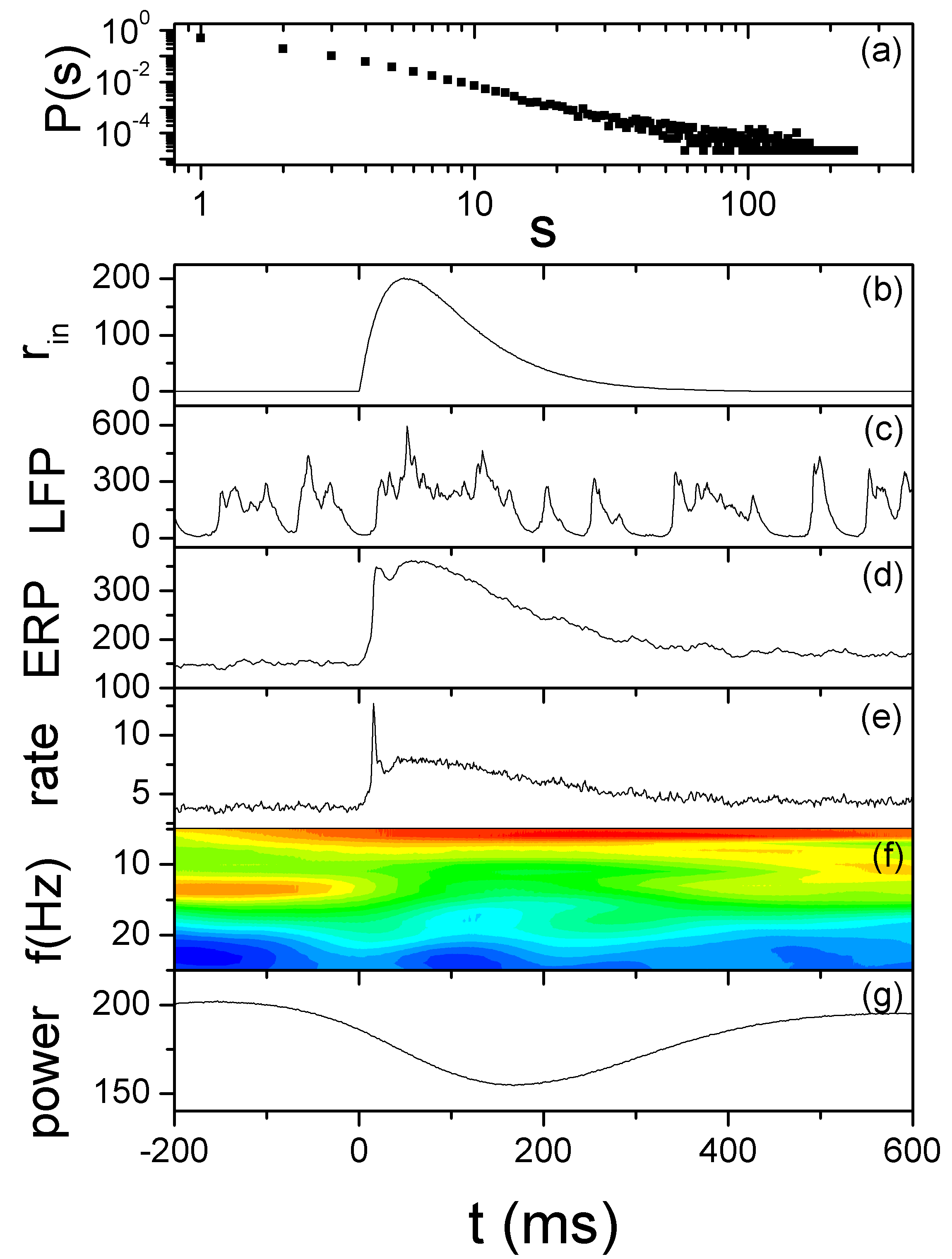


**S2 Fig. The Modular Network.** The ongoing activity and response of modular network which consists of 20 modules. (a) The avalanche size distribution of one module. The result was obtained from the modular networks in the self-sustained activity state without background input. (b) Firing rate of input signal to one of the modules. (c) The LFP signal of the stimulated module. The stimuli were added at t=0. (d) The ERP signal in the stimulated module. (e) The firing rate of neurons in the stimulated module. (f) Time-frequency representation of the stimulated module’s LFP signal. (f) The power of alpha band (from 10 to 15 Hz). The results in (d-g) were averaged over 100 trials. The excitatory coupling strength $\Delta g_{ex}=$0.4, and the inhibitory coupling strength $\Delta g_{inh}=5.0$ in all simulations.
